# Sacubitril/Valsartan Ameliorates Crizotinib-Induced Cardiotoxicity in Mice

**DOI:** 10.31083/j.rcm2407192

**Published:** 2023-07-03

**Authors:** Lijun Cheng, Junying Duan, Gary Tse, Tong Liu, Guangping Li

**Affiliations:** ^1^Tianjin Key Laboratory of Ionic-Molecular Function of Cardiovascular Disease, Department of Cardiology, Tianjin Institute of Cardiology, The Second Hospital of Tianjin Medical University, 300211 Tianjin, China; ^2^Department of Health Sciences, School of Nursing and Health Studies, Hong Kong Metropolitan University, 518057 Hong Kong, China

**Keywords:** cardiotoxicity, crizotinb, sacubitril/valsartan, *Myh7*

## Abstract

**Background:**

Lung cancer is one of the major cause of death globally. Crizotinib is a first-line drug used in treating non-small-cell lung cancer (NSCLC). However, the pathophysiological mechanisms underlying its cardiotoxicity are unknown. This study investigated the mechanisms of crizotinib-induced cardiotoxicity and explored whether this toxicity can be prevented by the angiotensin receptor/neprilysin inhibitor sacubitril/valsartan.

**Methods:**

Male C57BL/6 mice were randomly divided into three groups: control, crizotinib (40 mg⋅${\mathrm{kg}}^{-1}$⋅$d^{-1}$ for four weeks), and crizotinib + sacubitril/valsartan (40 mg⋅${\mathrm{kg}}^{-1}$⋅$d^{-1}$/60 mg⋅${\mathrm{kg}}^{-1}$⋅$d^{-1}$ for four weeks). Expression of genes in myocardial tissue were detected by transcriptomic sequencing, with verification of the differentially expressed genes (DEGs) using Real time-polymerase chain reaction (RT-PCR). Blood pressure (BP) and cardiac function of animals were measured using non-invasive monitoring and echocardiography approaches. Ventricular refractory period (RP), as well as the induction rate and score of ventricular arrhythmias (VAs) were detected by *in vivo* electrophysiology. Epicardial conductance was measured by mapping. Expression of *Myh7* in myocardium was detected by western blot and RT-PCR.

**Results:**

DEGs detected using transcriptomic sequencing included 10 up-regulated and 20 down-regulated genes. The first 5 DEGs identified were *Myh7*, *Ngp*, *Lcn2*, *Ciart* and *Ptgds*. Kyoto Encyclopedia of Genes and Genomes (KEGG) result indicated that *Myh7* is involved in myocarditis, cardiomyopathy, and cardiac muscle contraction. Crizotinib treatment increased blood pressure, prolonged QTc interval, shortened ventricular RP, increased the incidence and score of right VAs, and increased *Myh7* expression. Most of these responses were limited by sacubitril/valsartan.

**Conclusions:**

Crizotinib induced a range of cardiotoxic side effects in a mouse model and increased *Myh7* expression represents a biomarker for this response. These cardiovascular toxic responses can be largely prevented by sacubitril/valsartan.

## 1. Introduction 

Lung cancer is one of the major cause of deaths globally. Non-small cell lung cancer (NSCLC) contributes to 80–85% of all lung cancer cases [1, 2, 3]. Rearrangements in the genes encoding for anaplastic lymphoma kinase (ALK) and v-ros UR2 sarcoma virus oncogene homolog 1 (ROS1) are observed in 2–7% and 1–2% of NSCLC samples, respectively [4, 5]. Crizotinib, an adenosine-triphosphate (ATP)-competitive small molecule inhibitor, was the first oral ALK inhibitor approved by the Food and Drug Administration (FDA) in August 2011 for the treatment of NSCLC to inhibit the receptor tyrosine kinases ALK, ROS1, and mesenchymal-epithelial transition (MET) [5, 6, 7, 8, 9]. Crizotinib can also be used to treat multiple myeloma [10, 11, 12] and, although crizotinib is prone to drug resistance with repeated use, it remains a promising option for the treatment of NSCLCs [13]. For ALK-positive NSCLC, crizotinib is more effective and better tolerated than chemotherapy [14, 15]. In NSCLC with ROS1 rearrangement, crizotinib can be used as first-line treatment [5, 6, 7, 8]; however, cardiotoxicity caused by different anti-cancer drugs has long been recognized [11]. Previous studies showed that crizotinib induces various cardiotoxicities such as bradycardia, QT prolongation, ventricular rhythm, and ventricular fibrillation [16, 17, 18]. Among these side effects, the most common reports are that crizotinib prolongs the QT interval and reduces heart rate [19, 20, 21]. Thus, patients receiving crizotinib should receive close and regular monitoring of both the QT interval and heart rate [20]. Early identification of the cardiotoxicities associated with crizotinib is conducive to rational drug use; however, the specific mechanism(s) underlying crizotinib cardiotoxicity remain unclear.

To avoid, or reduce, cardiotoxicity associated with anti-cancer drugs, the administration of cardioprotective agents is critical. For example, prophylactic administration of a renin-angiotensin system (RAS) antagonist partially attenuates the cardiotoxic effects of doxorubicin in a chronic mouse model of chemotherapy-induced cardiac insufficiency [22]. Left ventricular ejection fraction (LVEF) was increased, and troponin I (TnI) was decreased, during a 6-month follow-up period of anthracycline treatment combined with carvedilol, suggesting a protective effect for carvedilol against myocardial injury [23]. The angiotensin receptor/neprilysin inhibitor sacubitril/valsartan is also used to treat heart failure and hypertension [24, 25, 26]. Previous studies using either animal models or human clinical trials showed that sacubitril/valsartan reversed cardiac remodeling, modulated heart failure biomarkers, reduced arrhythmias, improved renal function, improved the quality of life, and reduced mortality and/or the risk of hospitalization [27, 28, 29, 30]. Sacubitril/valsartan has also demonstrated utility in the treatment of cancer therapy-related cardiac dysfunction [31, 32] and with findings of improved cardiac function and cardiac-related symptoms [33]. In a study that combined sacubitril/valsartan and doxorubicin, sacubitril/valsartan was found to attenuate doxorubicin-induced apoptosis and endoplasmic reticulum stress in cultured H9C2 cardiomyocytes [34]. Similar findings were observed in a doxorubicin-induced rat cardiotoxicity model that examined biochemical markers, contractile function, endoplasmic reticulum stress, and attenuated doxorubicin-induced apoptosis in rat heart [35]. However, whether sacubitril/valsartan can reduce the cardiotoxicity induced by crizotinib, as well as the molecular nature of crizotinib induced cardiotoxicity remains unclear. Herein, we sought to investigate the effects of crizotinib on cardiotoxicity and determine whether sacubitril/valsartan can ameliorate crizotinib-induced cardiotoxicity.

## 2. Methods

### 2.1 Experimental Animal

This study was approved by the Laboratory Animal Ethical Committee of Chinese Academy Medical Sciences Institute of Radiation Medicine. A total of 36 male C57BL/6 mice were divided randomly into three groups: control (CON group), crizotinib (CRI group) and crizotinib + sacubitril/valsartan group (CRI + SV group). Mice in the crizotinib group were administered 40 mg⋅${\mathrm{kg}}^{-1}$⋅$d^{-1}$ crizotinib, dissolved in dimethyl sulfoxide (DMSO), for four consecutive weeks. Mice in the crizotinib + sacubitril/valsartan group were administered 40 mg⋅${\mathrm{kg}}^{-1}$⋅$d^{-1}$ crizotinib and 60 mg⋅${\mathrm{kg}}^{-1}$⋅$d^{-1}$ sacubitril/valsartan (also dissolved in DMSO). Cardiography, mapping, and cardiac electrophysiology *in vivo* were conducted, and myocardial tissue was dissected after sacrifice for subsequent experiments.

### 2.2 BP Measurement in Mice

Conscious animals were pre-warmed in a warm-up chamber at 36 °C–37 °C for 15 mins and their systolic, diastolic and mean arterial blood pressure (SBP, DBP and MBP, respectively) were recorded by using tail sleeve plethysmography (BP98AL, Softron, Tokyo, Japan).

### 2.3 Echocardiography

After weighing mice, chest hair was removed with a hair removal cream, followed by anesthesia with 2% isoflurane. Transthoracic echocardiography was performed using Imaging System (Vevo 2100, VisualSonics, Toronto, Canada). Data collected included left atrial diameter (LAD), left ventricular diameter at systolic and diastolic period (LVIDs and LVIDd, respectively), left ventricular anterior wall thickness at systolic and diastolic period (LVAWs and LVAWd, respectively), left ventricular posterior wall thickness at systolic and diastolic period (LVPWs and LVPWd, respectively), interventricular septum thickness at systolic and diastolic period (IVSs and IVSd, respectively), pulmonary artery acceleration time (PAT), left ventricular fractional shortening (FS), and left ventricular ejection fraction (EF).

### 2.4 Mapping

Using mice anesthetized with 1.5% tribromoethanol (0.02 mL/g; WXBD3759V, Sigma, St. Louis, Missouri, USA), and supported by a tracheal intubation ventilator, mouse chests were surgically opened with full exposure to the heart. Following this, the pericardium was removed, epicardial conduction velocity (CV), absolute inhomogeneity and inhomogeneity index was recorded and analyzed using the Electrical Mapping System (EMS64-USB-1003, MappingLab, Oxford, UK) and EMapScope 4.0 (MappingLab, Oxford, UK), as detailed previously [36].

### 2.5 Cardiac Electrophysiology in vivo

A programmed electrical stimulation protocol was performed using electrodes on the epicardial surface of the right ventricle (RV) and left ventricle (LV). The stimulation was performed at eight beats (120 ms, 8 × S1), followed by one extrastimulus (S2). The S1S2 interval gradually narrowed until a refractory period (RP) of RV and LV appeared. The stimulation was performed at eight beats (120 ms, 8 × S1), followed by one to three extra stimuli (S2, S3, and S4). The stimulation method was used to detect the ventricular arrhythmia score (VAs). At the same time, RV and LV was stimulated by burst (4 V, 20 Hz, 5 s) to detect the induction rate of VAs. The experimental protocols were typically completed within 20 mins [37].

### 2.6 The Transcriptome Sequencing and Bioinformatics Analysis

RNA extraction, transcriptome sequencing, and data analysis was performed by OE Biotech Co., Ltd. (Shanghai, China). In brief, total myocardial tissue RNA from myocardial in control (n = 3) and crizotinib groups (n = 3) was isolated by the mirVana™ miRNA ISOlation Kit (AM1561, Ambion, Austin, TX, USA). Following this, synthesis, purification and adapter ligation of cDNA was carried out. DNA libraries were created using TruSeq Stranded mRNA LTSample Prep Kit (NR604-02, Illumina, San Diego, CA, USA). The quality of libraries was assessed using an Agilent 2100 Bioanalyzer (2100, Agilent, Santa Clara, CA, USA). DNA libraries were sequenced using an Illumina sequencing platform (Nova6000, Illumina, San Diego, CA, USA).

*p* value < 0.05 and fold change (FC) >2 or FC <0.5 were used as the thresholds for screening for differentially expressed genes (DEGs). Hierarchical cluster analysis of DEGs was carried out to investigate DEGs expression pattern. A volcanic map of DEGs was drawn to understand the overall distribution of differential genes. Gene ontology (GO) enrichment and Kyoto encyclopedia of genes and genomes (KEGG) enrichment analysis of DEGs were performed to determine the biological function or pathways impacted by DEGs.

### 2.7 Myocardial Histopathology 

Mouse ventricular tissue was perfused with 10% neutral buffered formalin for 72 h at room temperature. These tissues were then dehydrated with an ethanol at different concentrations, followed by xylene and finally paraffin embedding and storage at –20 °C overnight. Embedded tissue was cut into 4 µm thick sections and hematoxylin and eosin (HE) (20220211, Solarbio, Beijing, China) staining was conducted to observe whether there are any changes in the arrangement or size of the cardiomyocyte nuclei. Masson Tricolor Staining (20220214, Solarbio, Beijing, China) was used to observe whether myocardial tissue was fibrotic.

### 2.8 Real Time-Polymerase Chain Reaction (RT-PCR)

RNA extraction (0000458714, Promega, Beijing, China) followed by reverse transcription of RNA into cDNA was conducted using a reverse transcription kit (X0222, Tiangen, Beijing, China). Subsequently, RT-qPCR was conducted using SYBR green (P31221, TransGen, Beijing, China) and a Quant Gene 9600 System (9600, Bioer Technology, Hangzhou, China). The $2^{-\Delta \,\Delta \,\text{CT}}$ method was used to obtain relative mRNA levels. Primers used for RT-PCR are detailed in Table 1.

**Table 1.** **Primer sequences**.

Gene name		Primer sequence
*Myh7*	forward	GACAGGAAGAACCTACTGCG
	reverse	GAACTTGGACAGGTTGGTGT
*Ciart*	forward	AGTGAAGAAGCTGCATACCG
	reverse	CAGCTCCCGTAGTACCAAAG
*Ngp*	forward	GAGGCCCTTCGACAACTAAG
	reverse	TTCTGACTAGAAGGCGGAGT
*Lcn2*	forward	TGACAACTGAATGGGTGGTG
	reverse	GATGCTCCTTGGTATGGTGG
*Ptgds*	forward	CTCCTTCTGCCCAGTTTTCC
	reverse	AATCCCAAGAGACCCAGGAG

*Myh7*, myosin, heavy polypeptide 7, cardiac muscle, beta; *Ciart*, circadian associated repressor of transcription; *Ngp*, neutrophilic granule protein; *Lcn2*, lipocalin 2; *Ptgds*, prostaglandin D2 synthase.

### 2.9 Western Blot

Total tissue protein was isolated by RIPA buffer (01408/15322, Cwbio, Beijing, China) and PMSF protease inhibitor (01392/06122, Cwbio, Beijing, China). Protein concentrations were measured using a bicinchoninic acid (BCA) Protein Concentration Assay Kit. The protein samples were separated on an 8% sodium dodecyl sulfate polyacrylamide gel electrophoresis (SDS-PAGE) gel and subsequently transferred to a polyvinylidene fluoride (PVDF) membrane. The PVDF membrane was blocked with 5% milk, subsequent incubation with primary β-actin antibody (1:5000, F210074, Proteintech, Wuhan, China) and Myh7 (1:1000, 00101476, Proteintech, Wuhan, China) primary antibody at 4 °C overnight. Following this, the membranes were washed with TBST, and incubated with goat anti-rabbit secondary antibody (1:5000) for one hour at room temperature, the color developer Rhea ECL (LEK22118, Life-iLab, Shanghai, China) was used to develop the western blots and band intensity was analyzed using Image Lab software and relative protein abundance computed using β-actin as the internal standard.

### 2.10 Statistical Analysis

The data analysis was carried out using Origin 6.0 (OriginLab, Northampton, MA, USA) and SPSS 17.0 (SPSS Inc., Chicago, IL, USA) software. The results are presented are expressed as mean ± standard deviation (SD). A one-way ANOVA was used to compare the groups and least significant difference (LSD)-*t *test was conducted for post-hoc analysis. Statistically significant results were defined as having a *p*-value of less than 0.05.

## 3. Results

### 3.1 Effects of Crizotinib on Blood Pressure, Pathology, and Epicardial Electrical Conduction

**Fig. 1.**
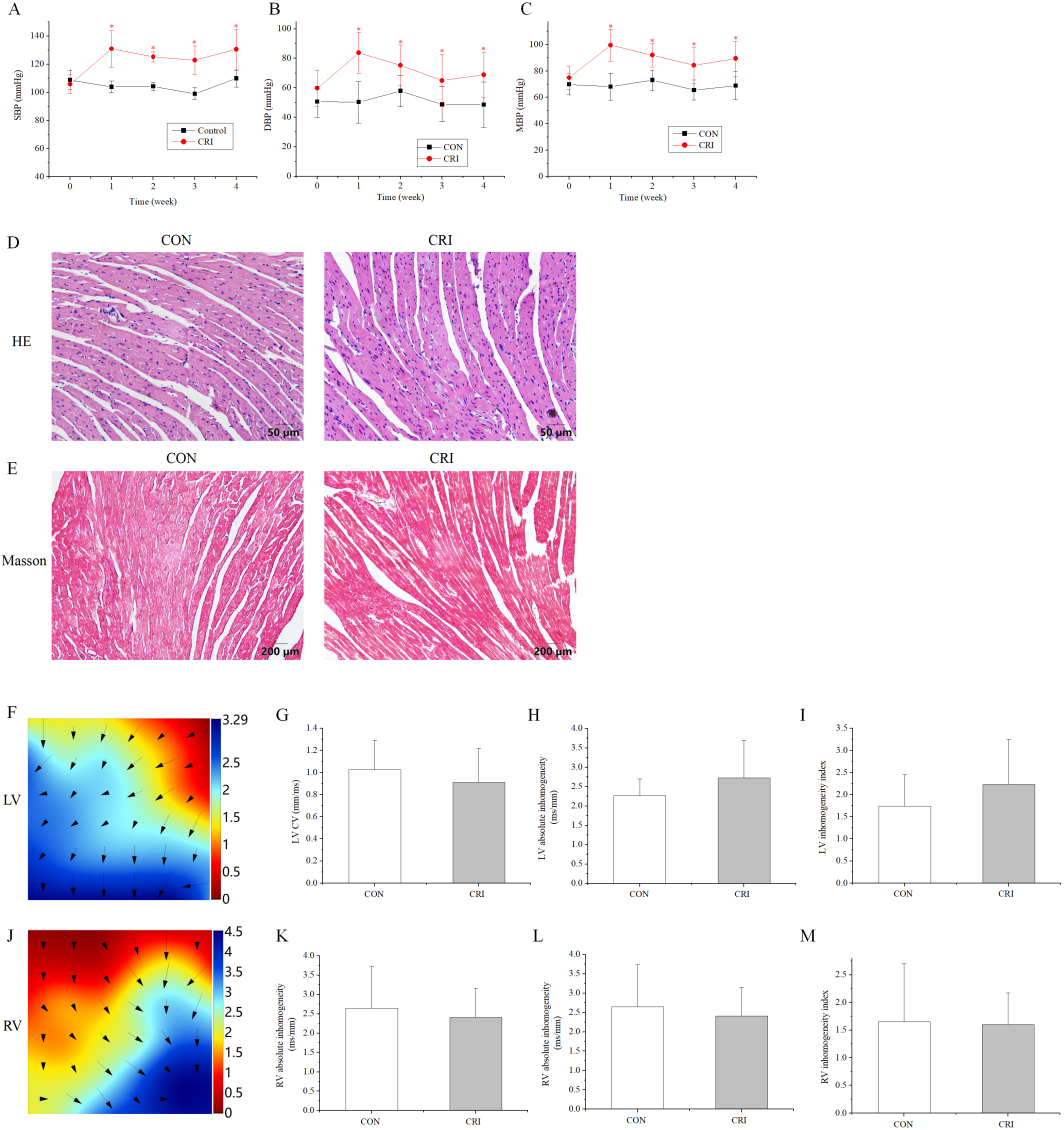
**Effect of crizotinib on BP, myocardial pathology, and electrical conduction characteristics in control and crizotinib group mice**. (A) Effects of crizotinib on SBP. (B) Effects of crizotinib on DBP. (C) Effects of crizotinib on MBP. (D) Typical sample of HE staining in control and crizotinib group. (E) Typical sample of Masson staining in control and crizotinib group. (F) Representative epicardial electrical mapping of recorded LV. (G) CV of LV. (H) Absolute inhomogeneity of LV. (I) Inhomogeneity index of LV. (J) Representative epicardial electrical mapping recording of RV. (K) CV of RV. (L) Absolute inhomogeneity of RV. (M) Inhomogeneity index of RV. *p < 0.05 *vs* CON group. CON, control group; CRI, crizotinib group; BP, blood pressure; SBP, DBP and MBP, systolic, diastolic and mean arterial blood pressure respectively; LV, left ventricular; RV, right ventricular; CV, conduction velocity; HE, hematoxylin and eosin.

To study the cardiotoxicity caused by crizotinib, the blood pressure (BP) of mice after crizotinib administration was measured. The SBP, DBP and MBP were significantly higher following crizotinib treatment for 1 week (n = 10, SBP, *p* = 0.000; DBP, *p* = 0.000; MBP, *p* = 0.000), and remained significantly elevated at 4 weeks after treatment (n = 10, SBP, *p* = 0.000; DBP, *p* = 0.009; MBP, *p* = 0.001) when compared to the control group (Fig. 1A–C). HE and Masson staining was performed on ventricular muscle tissue obtained from mice 4 weeks after crizotinib use. No significant changes in myocardial tissue structure nor significant myocardial fibrosis was observed in control and crizotinib group mice (n = 5) (Fig. 1D,E). We also recorded the characteristics of epicardial electrical conduction of mice using mapping. Fig. 1F is a representative epicardial electrical mapping of LV. The calculated CV (n = 5, *p* = 0.553), absolute inhomogeneity (n = 5, *p* = 0.365), and inhomogeneity index (n = 5, *p* = 0.404) of LV were not significantly different between the control and crizotinib groups (Fig. 1G–I). A representative epicardial electrical mapping of RV is shown in Fig. 1J. The calculated CV (n = 5, *p* = 0.532), absolute inhomogeneity (n = 5, *p* = 0.702), and inhomogeneity index (n = 5, *p* = 0.926) of RV was also found to not change significantly in control and crizotinib groups (Fig. 1K–M).

### 3.2 Effects of Crizotinib on Myocardial Transcriptomics and Validation of Gene Expression

**Fig. 2.**
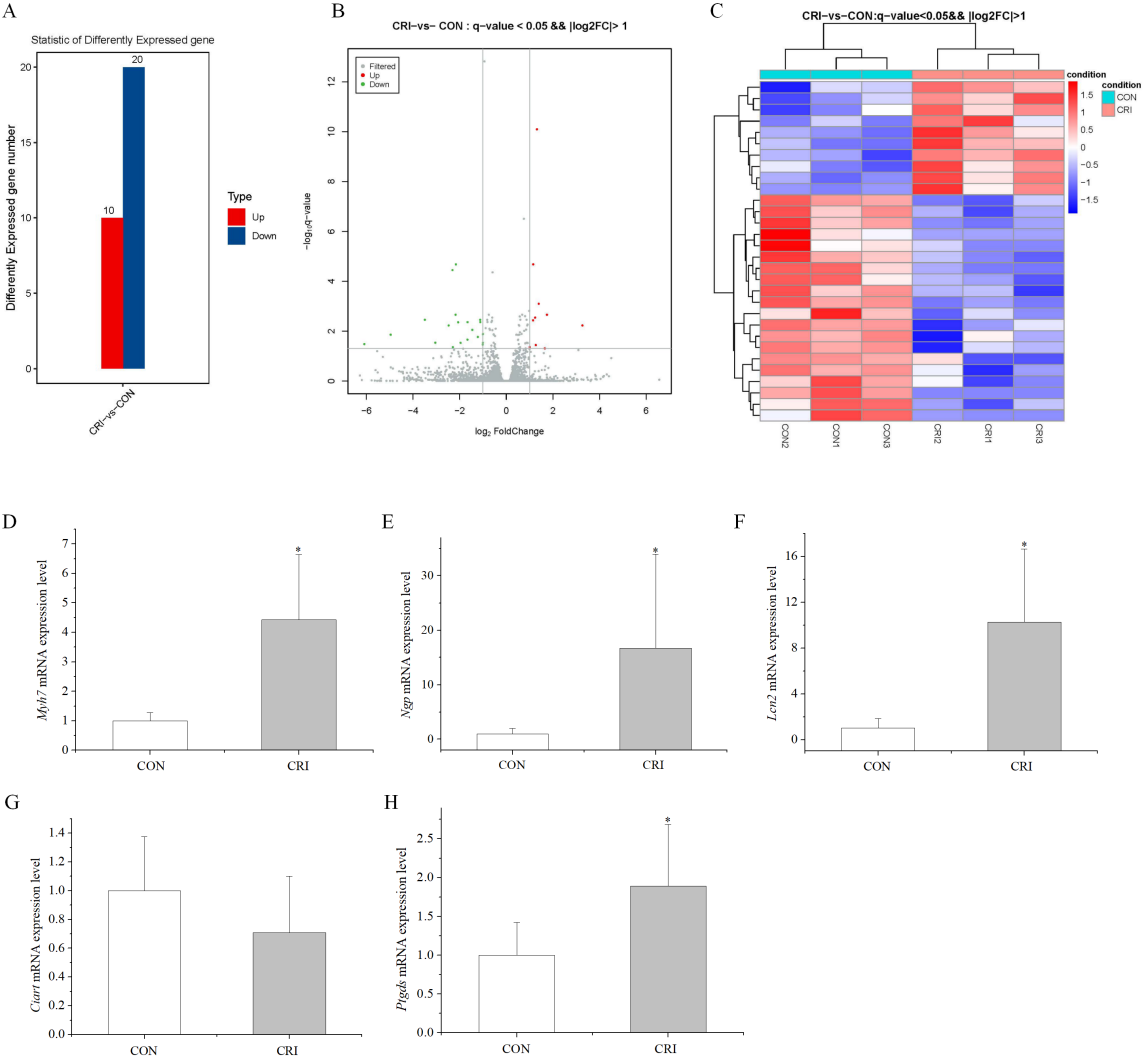
**Characteristics of DEGs**. (A) 30 DEGs identified in the crizotinib group, including 10 up-regulated and 20 down-regulated genes (the ordinate is differently expressed up-regulated and down-regulated gene number). (B) Volcano map of DEGs (the abscissa is $\log_{2}$ (FC)). The ordinate is ${\mathrm{–log}}_{10}$ (q-value). The green, red and gray dots indicate down-regulated DEGs, up-regulated DEGs and non-significantly regulated genes, respectively. (C) Cluster heatmap of the 30 DEGs. Red and blue indicates high and low expression genes respectively. (D–H) Validation of *Myh7*, *Ngp*, *Lcn2*, *Ciart* and *Ptgds* by Real time-polymerase chain reaction (RT-PCR). Three samples per group for myocardial transcriptomics experiments, and five samples per group for RT-PCR experiments. *p < 0.05 *vs* CON group. CON, control group; CRI, crizotinib group; DEGs, differentially expressed genes; *Myh7*, myosin, heavy polypeptide 7, cardiac muscle, beta; *Ciart*, circadian associated repressor of transcription; *Ngp*, neutrophilic granule protein; *Lcn2*, lipocalin 2; *Ptgds*, prostaglandin D2 synthase.

To screen for alterations in gene expression following crizotinib treatment of myocardial tissue, we performed transcriptomic analysis on control and crizotinib group mice. Three mouse myocardial tissue samples in each group were analyzed. *p* value < 0.05 and FC >2 or FC <0.5 were used as the threshold for establishing DEGs. Compared with controls, there were 30 DEGs identified in the crizotinib group mice, these included 10 up-regulated and 20 down-regulated genes (Fig. 2A). Fig. 2B shows the the DEGs using a volcano map. Fig. 2C shows a cluster heatmap of DEGs.

Among the DEGs, we were most interested in highly expressed genes after crizotinib exposure. This led us to select the top five differentially up-regulated genes for validation using RT-PCR (primer sequences given in Table 1). These genes included *Myh7* (myosin, heavy polypeptide 7, cardiac muscle, beta), *Ciart* (circadian associated repressor of transcription), *Ngp* (neutrophilic granule protein), *Lcn2* (lipocalin 2), and *Ptgds* (prostaglandin D2 synthase). RT-PCR results showed that 4 of these 5 genes displayed significantly increased expression in crizotinib mice (n = 5, *Myh7*, *p* = 0.007; *Ngp*, *p* = 0.015; *Lcn2*, *p* = 0.011; *Ciart*, *p* = 0.245; *Ptgds*, *p* = 0.016), which was in good agreement with the results of transcriptomic analysis except for *Ciart* (Fig. 2D–H).

### 3.3 GO/KEGG Analysis of DEGs and Screening of Key Genes

**Fig. 3.**
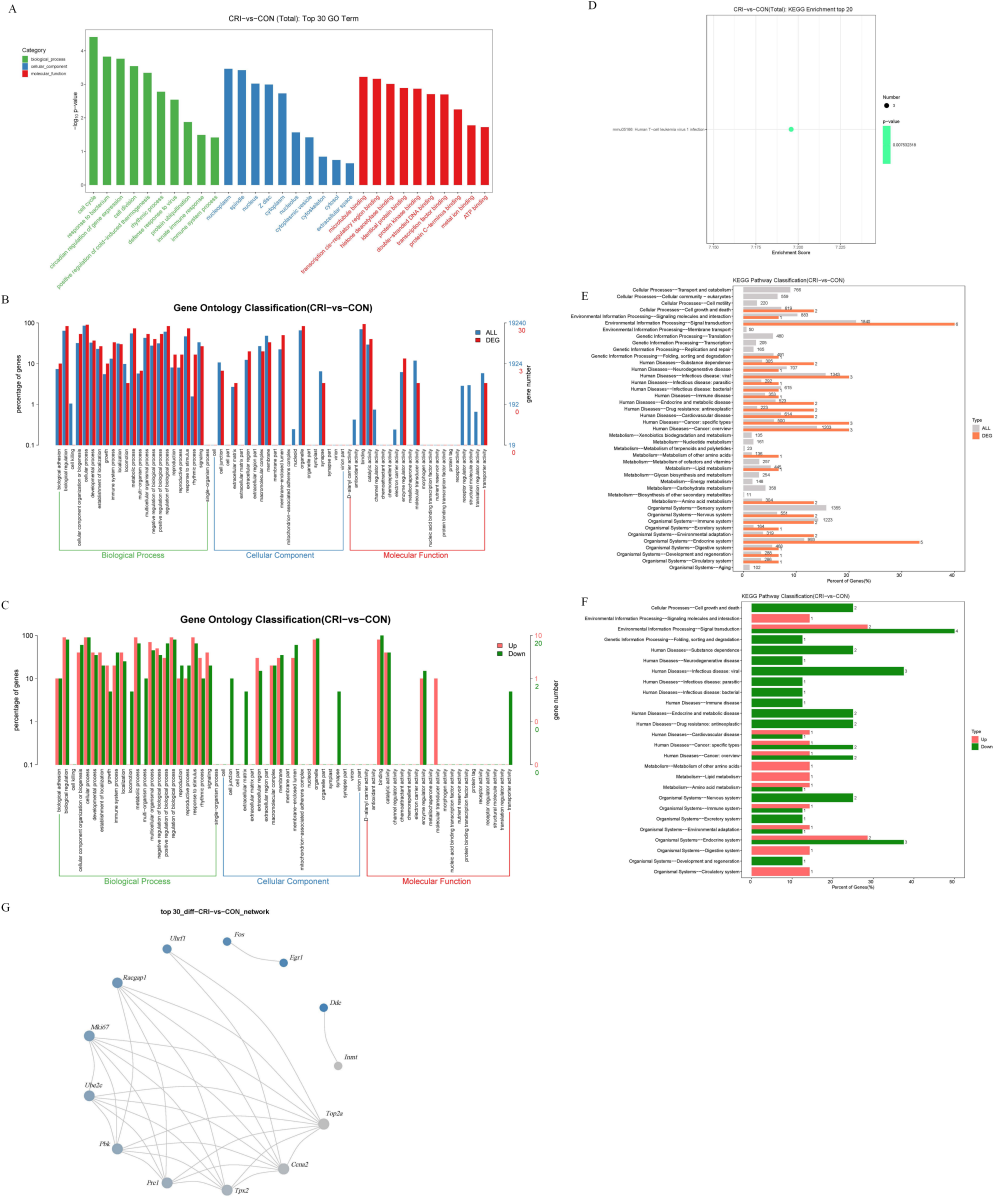
**GO and KEGG analysis of DEGs**. (A) Top 30 GO terms. The abscissa is the GO term, the ordinate is ${\mathrm{–log}}_{10}$ (*p*-value). (B) Comparative distribution of all genes and DEGs at GO level 2. The abscissa is the GO term, the ordinate is the number and its percentage of genes. (C) Comparative distribution of up and down-regulated DEGs at GO level 2. The abscissa is the GO term, ordinate is the number and its percentage of genes. (D) KEGG enrichment top 20 identified DEGs. The abscissa is the enrichment score, and the ordinate is the pathway information. (E) The distribution of all genes and DEGs at KEGG level 2. The abscissa is the number and ratio (%) of all genes and DEGs. The ordinate is the name of the pathway. (F) The distribution of up and down-regulated DEGs at KEGG level 2. The abscissa is the number and ratio (%) of up and down-regulated DEGs. The ordinate is the name of the pathway. (G) Gene interaction network showing interaction between DEGs. CON, control group; CRI, crizotinib group; DEGs, differentially expressed genes; KEGG, Kyoto Encyclopedia of Genes and Genomes; GO, gene ontology; ATP, adenosine-triphosphate; *Fos*, FBJ osteosarcoma oncogene; *Uhrf1*, ubiquitin-like, containing PHD and RING finger domains, 1; *Racgap1*, Rac GTPase-activating protein 1; *Mki67*, antigen identified by monoclonal antibody Ki 67; *Ube2c*, ubiquitin-conjugating enzyme E2C; *Pbk*, PDZ binding kinase; *Prc1*, protein regulator of cytokinesis 1; *Tpx2*, microtubule-associated; *Ccna2*, cyclin A2; *Top2a*, topoisomerase (DNA) II alpha; *Inmt*, indolethylamine N-methyltransferase; *Ddc*, dopa decarboxylase; *Egr1*, early growth response 1.

Following identification of DEGs, we next analyzed these genes using GO/KEGG to understand their functions. These genes were grouped into categories according to their characteristics in “biological process”, “cellular component”, and “molecular function”. The top 3 GO terms for “biological process” were “cell cycle”, “response to bacterium”, and “circadian regulation of gene expression”. The top 3 GO terms for “cellular component” were “nucleoplasm”, “spindle”, and “nucleus”. The top 3 GO terms for “molecular function” were “microtubule binding”, “transcription cis-regulatory region binding”, and “histone deacetylase binding” (Fig. 3A). According to the assigned characteristics of “biological process”, “cell composition” and “molecular function” of these genes, their level 2 function was graded. The functional distribution of all DEGs at GO Level 2 is shown in Fig. 3B. The functional distributions of differentially up-regulated and down-regulated genes at GO Level 2 is shown in Fig. 3C.

KEGG analysis was performed on the identified DEGs to systematically analyze the their regulatory role. KEGG enrichment of the top 20 identified genes is shown in Fig. 3D, the roles only include the term “human T-cell leukemia virus 1 infection”. Further, the distributions of all genes and DEGs at KEGG Level 2 is displayed in Fig. 3E. The distributions of up and down-regulated DEGs at KEGG Level 2 are shown in Fig. 3F. Finally, we investigated the interaction relationship between DEGs using the STRING database (Fig. 3G). This analysis of gene interactions showed that one gene interacts directly or indirectly with another or more other genes.

Among the DEGs, we paid close attention to those related to human cardiovascular diseases. We screened 7 items in KEGG enrichment by classification_level1 ‘human disease’ or ‘organismal Systems’ and classification_level2 ‘cardiovascular diseases’ or ‘circulatory system’ (Table 2). This analysis indicated that *Myh7* is not only highly expressed, but also involved in multiple processes in KEGG enrichment. *Myh7* is closely related to a variety of cardiomyopathies, myocardial contraction, and adrenergic signaling in cardiomyocytes. Therefore, *Myh7* may be a potential gene target associated with crizotinib-induced cardiotoxicity.

**Table 2.** **KEGG enrichmen related to cardiovascular diseases**.

ID	Term	Classification_level1	Classification_level2	*p *values	GeneID
mmu05416	Viral myocarditis	Human diseases	Cardiovascular disease	0.132	*Myh7*
mmu04260	Cardiac muscle contraction	Organismal systems	Circulatory system	0.135	*Myh7*
mmu05410	Hypertrophic cardiomyopathy	Human diseases	Cardiovascular disease	0.147	*Myh7*
mmu05414	Dilated cardiomyopathy	Human diseases	Cardiovascular disease	0.152	*Myh7*
mmu04261	Adrenergic signaling in cardiomyocytes	Organismal systems	Circulatory system	0.234	*Myh7*
mmu05418	Fluid shear stress and atherosclerosis	Human diseases	Cardiovascular disease	0.227	*Fos*
mmu05417	Lipid and atherosclerosis	Human diseases	Cardiovascular disease	0.316	*Fos*

KEGG, Kyoto Encyclopedia of Genes and Genomes; *Myh7*, myosin, heavy polypeptide 7, cardiac muscle, beta; *Fos*, FBJ osteosarcoma oncogene.

### 3.4 Effects of Crizotinib and Sacubitril/Valsartan on Blood Pressure and Cardiac Function

**Fig. 4.**
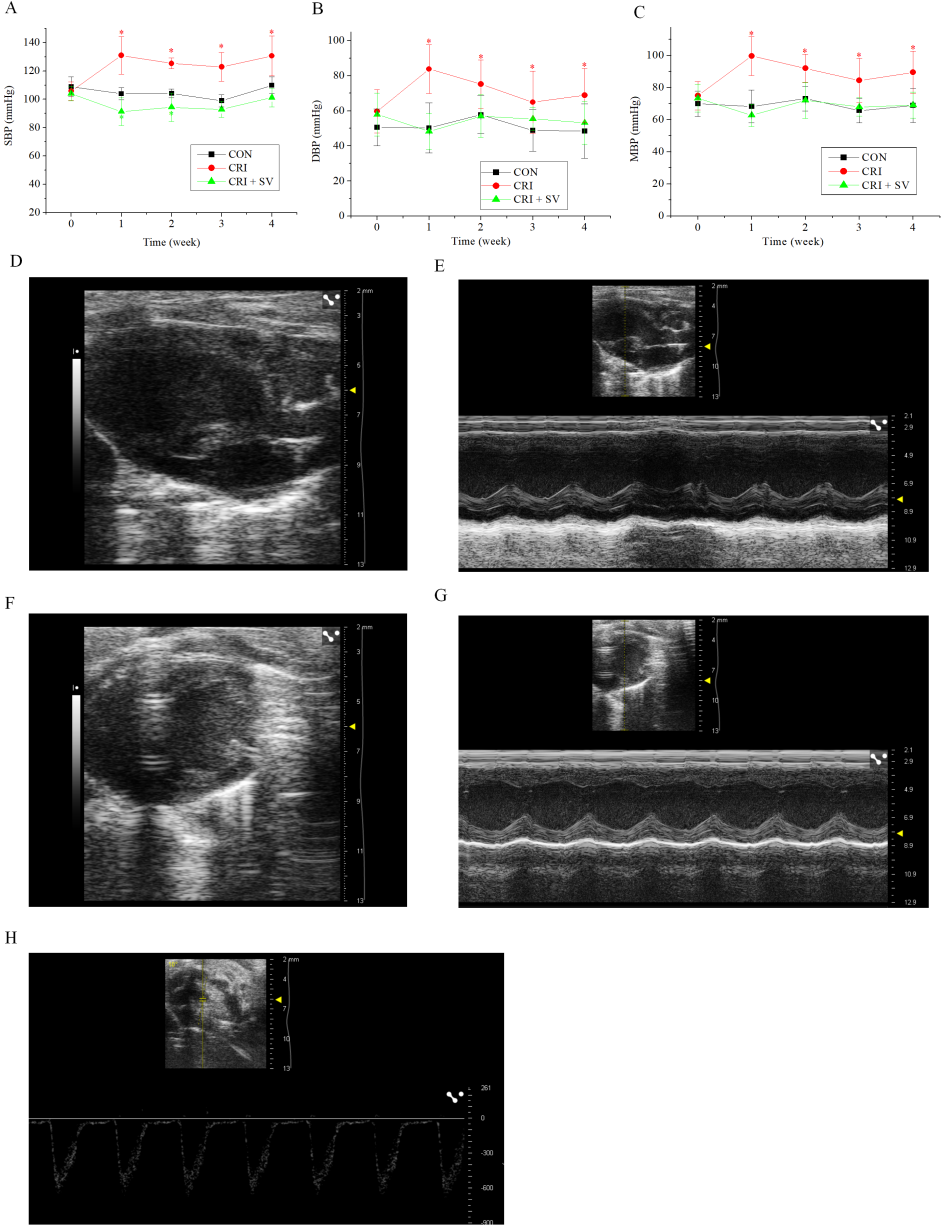
**Effect of crizotinib and sacubitril/valsartan on BP and cardiac function in control, crizotinib, and crizotinib + sacubitril/valsartan mouse groups**. (A) Effects of crizotinib and sacubitril/valsartan on SBP. (B) Effects of crizotinib and sacubitril/valsartan on DBP. (C) Effects of crizotinib and sacubitril/valsartan on MBP. (D,E) B- and M-type echocardiogram long-axis view of the parasternal LV. (F,G) B- and M-type echocardiogram short-axis view. (H) Doppler pulse wave of pulmonary valve flow. *p < 0.05 *vs* CON group. CON, control group; CRI, crizotinib group; CRI + SV, crizotinib + sacubitril/valsartan group; BP, blood pressure; SBP, DBP and MBP, systolic, diastolic and mean arterial blood pressure respectively.

In view of the above results, crizotinib cardiotoxicity appears to be principally manifested as an increase in BP. Sacubitril/valsartan is a commonly used drug for BP reduction, thus we added an additional animal group to our study composed of crizotinib combined with sacubitril/valsartan, this was termed the crizotinib + sacubitril/valsartan group. The SBP, DBP and MBP line charts of the three experimental groups are shown in Fig. 4A–C. It can be seen that that the rise of SBP, DBP and MBP was caused by crizotinib at different timepoints (n = 10). However, data gathered indicate that sacubitril/valsartan given in combination with crizotinib can reduce elevated BP at 4 weeks when compared with the control group, SBP, *p* = 0.054; DBP, *p* = 0.473; MBP, *p* = 0.967.

To observe the effect of the crizotinib + sacubitril/valsartan combination on cardiac function we recorded the echocardiography of control, crizotinib, and crizotinib + sacubitril/valsartan groups. Fig. 4D–H shows typical images of the parasternal LV long-axis view. Also shown are B-type and M-type echocardiograms of long and short-axis views, and doppler pulse wave of pulmonary valve flow. Echocardiographic parameters of these three groups are shown in Table 3. The results indicated that there was no significant difference in IVSd, LVPWs, LVPWd, LVIDs, LVIDd, LVAWs, LVAWd, FS, EF or LAD among the three groups (n = 10). However, we found that the PAT of crizotinib group increased and the IVSs decreased when compared with the control group (n = 10). However, these two values returned to the level of the control group after crizotinib was combined with sacubitril/valsartan (n = 10).

**Table 3.** **Echocardiographic parameters**.

	CON (n = 10)	CRI (n = 10)	CRI + SV (n = 10)	*p* values
PAT (ms)	15.97 ± 2.56	20.39 ± 2.38*	15.53 ± 2.67	0.000
IVS;s (mm)	1.37 ± 0.33	1.09 ± 0.23*	1.32 ± 0.16	0.045
IVS;d (mm)	0.83 ± 0.17	0.72 ± 0.10	0.78 ± 0.12	0.198
LVPW;s (mm)	1.41 ± 0.20	1.38 ± 0.14	1.42 ± 0.26	0.933
LVPW;d (mm)	0.96 ± 0.11	0.92 ± 0.15	0.94 ± 0.18	0.894
LVID;s (mm)	1.94 ± 0.50	2.04 ± 0.31	2.03 ± 0.35	0.847
LVID;d (mm)	3.30 ± 0.21	3.26 ± 0.18	3.30 ± 0.36	0.910
LVAW;s (mm)	1.35 ± 0.23	1.33 ± 0.21	1.29 ± 0.21	0.793
LVAW;d (mm)	0.80 ± 0.13	0.90 ± 0.16	0.77 ± 0.13	0.113
FS (%)	41.55 ± 13.05	37.72 ± 7.10	39.00 ± 5.78	0.642
EF (%)	71.53 ± 13.96	68.47 ± 8.96	70.25 ± 7.09	0.806
LAD (mm)	2.34 ± 0.3	2.39 ± 0.22	2.38 ± 0.48	0.954

*p < 0.05 *vs* CON group. CON, control group; CRI, crizotinib group; CRI + SV, crizotinib + sacubitril/valsartan group; PAT, pulmonary artery acceleration time; LAD, left atrial diameter; FS, fractional shortening; LVID, left ventricular diameter; LVAW, left ventricular anterior wall thickness; LVPW, left ventricular posterior wall thickness; IVS, interventricular septum thickness; EF, ejection fraction.

### 3.5 Effects of Crizotinib and Sacubitril/Valsartan on Cardiac Electrophysiological Properties 

**Fig. 5.**
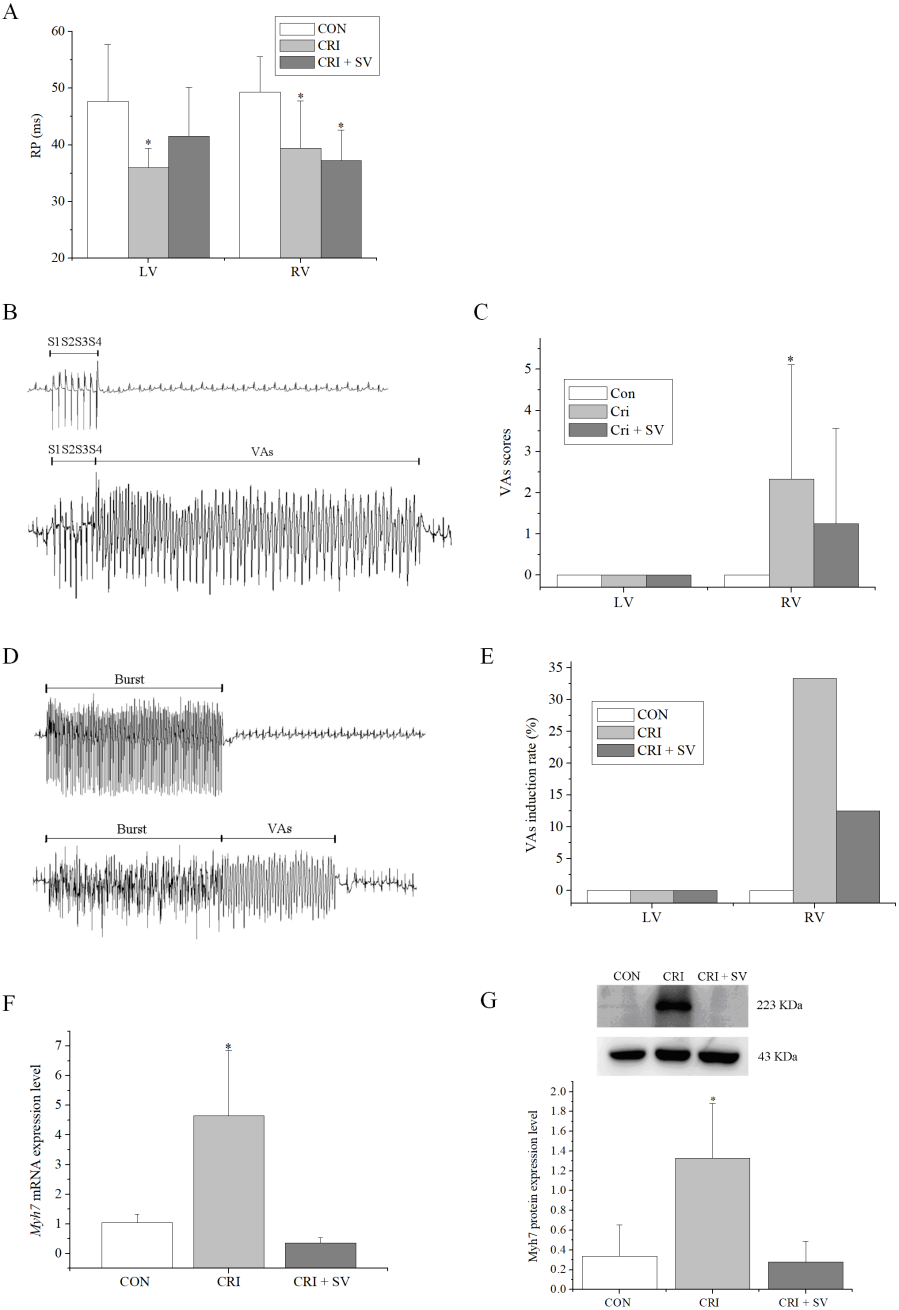
**Effects of crizotinib and sacubitril/valsartan on ventricular electrophysiology in control, crizotinib, and crizotinib + sacubitril/valsartan groups**. (A) Effects of crizotinib and sacubitril/valsartan on ventricular RP. (B) Typical examples of VAs occurring after 8 S1 stimulation followed by one to three extra stimuli (S2, S3, and S4). (C) Effect of crizotinib and sacubitril/valsartan on VAs score. (D) Typical examples of VAs occurring after burst stimulation. (E) Effect of crizotinib and sacubitril/valsartan on VAs induction rate after burst stimulation. (F) Effects of crizotinib and sacubitril/valsartan on *Myh7* mRNA expression. (G) Effects of crizotinib and sacubitril/valsartan on *Myh7* protein expression. *p < 0.05 *vs *CON group. CON, control group; CRI, crizotinib group; CRI + SV, crizotinib + sacubitril/valsartan group; VAs, ventricular arrhythmias; RP, refractory period; LV, left ventricular; RV, right ventricular; *Myh7*, myosin, heavy polypeptide 7, cardiac muscle, beta.

The effects of combination crizotinib and sacubitril/valsartan on cardiac electrophysiology *in vivo* were explored. Analysis of ECGs showed that the crizotinib group displayed faster heart rates, shorter RR intervals, and longer QTc compared to the control group (Table 4). Moreover, these abnormalities were restored in the crizotinib + sacubitril/valsartan group. A programmed electrical stimulation protocol was performed by stimulating the epicardial surface of the LV and RV. The RPs of LV and RV in the control (n = 7), crizotinib (n = 9), and crizotinib + sacubitril/valsartan (n = 8) groups are shown in Fig. 5A. A typical example of VAs occurring after 8 S1 stimulation followed by one to three extra stimuli (S2, S3, and S4) is shown in Fig. 5B. The VAs scores of the three groups were calculated and are shown in Fig. 5C and a typical example of VAs induced by burst stimulation is shown in Fig. 5D. The effects of crizotinib and sacubitril/valsartan on VAs induction rate after burst stimulation in the three groups are given in Fig. 5E. These data showed that crizotinib reduced the RPs of the LV and RV (LV, *p* = 0.006; RV, *p* = 0.010), increased the VAs score (*p* = 0.045), and increased the induction rate in the RV. Most of these abnormalities were prevented in the crizotinib + sacubitril/valsartan group when compared with controls (LV RPs, *p* = 0.130; VAs score of RV, *p* = 0.280). The exception to this was the RV RPs (*p* = 0.003).

**Table 4.** **ECG parameters**.

	CON (n = 10)	CRI (n = 15)	CRI + SV (n = 10)	*p* values
BW (g)	25.18 ± 1.15	23.27 ± 1.83	23.74 ± 4.10	0.197
HR (bpm)	389.70 ± 83.76	459.13 ± 70.72*	401.80 ± 67.70	0.053
RR interval (ms)	160.20 ± 32.91	133.73 ± 21.80*	153.20 ± 25.98	0.046
PR interval (ms)	44.70 ± 8.14	36.00 ± 12.91	46.40 ± 6.06	0.030
QT interval (ms)	74.90 ± 10.89	81.73 ± 14.41	64.40 ± 14.82	0.014
QTc (s)	0.19 ± 0.02	0.22 ± 0.04*	0.16 ± 0.03	0.000

*p < 0.05 *vs* CON group. CON, control group; CRI, crizotinib group; CRI + SV, crizotinib + sacubitril/valsartan group; ECG, electrocardiogram; BW, body weight; HR, heart rate; RR, R wave-to-R wave; PR, P wave-to-R wave.

### 3.6 Effects of Crizotinib and Sacubitril/Valsartan on the Expression of Myh7 in Myocardial Tissue 

Finally, changes in the *Myh7* expression levels by crizotinib with or without sacubitril/valsartan were determined by western blot and RT-PCR (Fig. 5F,G). Crizotinib increased both the mRNA and protein abundance of *Myh7* in the myocardium (n = 5, mRNA, *p* = 0.001; protein, *p* = 0.000). This effect was blunted by the use of crizotinib combined with sacubitril/valsartan when compared to controls (n = 5, mRNA: *p *= 0.414; protein: *p* = 0.803).

## 4. Discussion

In this study we reported the cardiotoxic side effects associated with crizotinib exposure, including increased BP and prolonged QTc intervals. These were associated with increased right VAs scores and induction rates, and increased myocardial expression of *Myh7* which is the most frequently mutated gene in hypertrophic cardiomyopathy. Most of these abnormalities were limited by co-treatment with sacubitril/valsartan.

### 4.1 Crizotinib and its Cardiotoxicity

Lung cancer is one of the major contributor to deaths globally, with NSCLC accounting for a large proportion of the tumor burden [1, 2, 3]. Crizotinib is approved for the treatment of NSCLC cases in which rearrangements in the genes encoding for ALK, ROS1 and MET are found [5, 7, 8, 9, 38]. Approximately 2–7% and 1–2% of NSCLC samples show rearrangements in ALK and ROS1, respectively [5]. For ALK inhibition in NSCLC, crizotinib is more effective and better tolerated than chemotherapy [39, 40]. However, patients treated with crizotinib develop drug resistance, requiring the use of second-generation ALK inhibitors to overcome crizotinib resistance. ROS1 rearrangement defines a second molecular subgroup of NSCLC for which crizotinib is highly active [7], and crizotinib displays marked antitumor activity in patients with advanced NSCLC with ROS1 rearrangement. In NSCLC with ROS1 rearrangement, crizotinib can be used as first-line treatment [5, 6, 7, 8]. Currently, only crizotinib is used for Anaplastic Large Cell Lymphoma (ALCL), belonging to the first generation of this drug class [4]. In crizotinib phase I studies, 94 percent of patients displayed some degree of tumor shrinkage during the study. In a phase 3, open-label trial comparing the differences between crizotinib and chemotherapy, the median progression-free survival of crizotinib was significantly higher than that of chemotherapy [7]. Previous studies have confirmed that crizotinib is superior to pemetrexed cisplatin or carboplatin, and is associated with a reduction in the main symptoms associated with lung cancer including cough, pain, and dyspnea [6].

Cardiotoxicity caused by anti-cancer drugs, including hypertension, arrhythmias, QTc interval prolongation, and left ventricular systolic dysfunction have long been a focus of attention [11]. Previous studies have demonstrated crizotinib-related cardiotoxicities of QT prolongation, bradycardia, ventricular fibrillation, and ventricular tachycardia [16]. In NSCLC, crizotinib was found to cause adverse cardiovascular side effects such as bradycardia, QT interval prolongation, ventricular rhythm, ventricular fibrillation, and pericarditis [17]. Another study reported QT interval prolongation, mild motion wall abnormalities in the left anterior wall and chamber door, small amounts of pericardial effusion, and even transient ventricular tachycardia and ventricular fibrillation [41]. In our mouse study, crizotinib exposure led to side effects such as increased BP, prolonged QTc, and inducible ventricular arrhythmias. We also observed a significant prolongation in PAT in the crizotinib group, suggesting that increased pulmonary artery pressure may cause right ventricular dysfunction in mice [42], whereas long QT interval is mainly associated with impaired ventricular function and cardiac exhaustion [43].

### 4.2 The Role of Myh7 in Crizotinib Induced Cardiotoxicity

Crizotinib can lead to increased caspase activation, cholesterol accumulation, and ion channel dysfunction [42]. Effective control of tumor growth can be achieved by dose-dependent inhibition of tyrosine phosphorylation of MET kinase and ALK [19]. After inhibition of 2-DIG-mediated glycolysis, crizotinib is inhibited by cell proliferation, migration, ATP production, mitochondrial transmembrane potential, or apoptosis signaling of mitochondria-associated cells. These findings suggest that crizotinib induces mitochondrial hypofunction and compensatory hyperoxic metabolism, without maintenance of adequate ATP levels. Moreover, the exchange pattern and inadequate supply of ATP may be an antitumor property of crizotinib [44]. Crizotinib is also a MET inhibitor, and MET has been implicated in cardiovascular remodeling after tissue injury as well as regulating mRNA levels of Glut4 and Ppars [45]. Further, the inhibition of potassium channels encoded by human ether-a-go-go (hERG)-related genes can lead to delayed repolarization, prolonged QT intervals, and life-threatening polymorphic ventricular tachycardia or Torsades de Pointes [46].

To further elucidate the mechanisms responsible for crizotinib-induced cardiotoxicity and to identify the genes that underlie its pathological effects, transcriptome sequencing of cardiac muscle tissue was used and this approach identified 10 up-regulated and 20 down-regulated genes in response to crizotinib exposure. Using GO and KEGG analysis of these DEGs, we selected terms directly related to cardiovascular disease and the circulatory system. *Myh7* is both highly expressed and involved in multiple processes in KEGG enrichment, including a variety of cardiomyopathy, myocardial contraction, and adrenergic signaling in cardiomyocytes. Therefore, *Myh7* may be a potential gene target of crizotinib-induced cardiotoxicity.

The *Myh7* gene encodes the beta myosin heavy chain subunit of cardiac myosin (beta-MHC). Changes in myosin expression can affect the contractile capacity of cardiomyocytes and lead to abnormal myocardial structure and/or function. Modification of myosin may affect the mechanical function of the myocardium and are therefore considered to be linked to myocardial dysfunction leading to heart failure [47]. To date, 186 and 73 β-*Myh7* gene mutations have been reported in cases of hypertrophic cardiomyopathy and dilated cardiomyopathy (DCM), respectively [48]. *Myh7* is predominantly expressed in the embryonic heart and is rarely expressed in adulthood. Myh7 pathogenic variants can cause a variety of cardiac diseases, including hypertrophic cardiomyopathy, DCM, left ventricular noncompaction cardiomyopathy, congenital fiber-type disproportion, and myosin myopathy [47]. *Myh7*-related DCM complications principally manifest as VAs and heart failure. Our results showed that with the occurrence of crizotinib-associated cardiac toxicity, the expression of *Myh7* in myocardium increased significantly suggesting that *Myh7* may be an important biomarker of crizotinib-induced cardiotoxicity.

### 4.3 Effect of Sacubitril/Valsartan on Improving Crizotinib Induced Cardiotoxicity

Sacubitril/valsartan is used clinically in hypertension and heart failure. It can reverse left ventricular hypertrophy and delays left ventricular remodeling. These effects reduce the risks of cardiovascular death or hospitalization, improve symptoms, in-hospital outcomes and mortality in patients with heart failure [49, 50]. Clinical practice guidelines classify sacubitril/valsartan as a Class I recommendation as an alternative to angiotensin converting enzyme inhibitor [51] and that sacubitril/valsartan is associated with a reduced incidence of VAs in heart failure with reduced ejection fraction (HFrEF) [52, 53]. In patients with nonischemic DCM, the use of sacubitril/valsartan can also improve ventricular function and clinical outcomes [54]. In regards to possible cardiac protection in cancer patients receiving anti-cancer therapies, the international guidelines for sacubitril/valsartan are less clear. However, an increasing body of evidence has reported the benefits of sacubitril/valsartan [24, 27] on, for example, doxorubicin-related cardiotoxicity [34]. Specifically, sacubitril/valsartan can limit doxorubicin-induced apoptosis and endoplasmic reticulum stress in cultured H9C2 cardiomyocytes and can improve biochemical markers, contractile function, and endoplasmic reticulum stress in a rat doxorubicin-induced cardiotoxicity model [35]. Indeed, the potential benefits of sacubitril/valsartan in patients with cancer treatment-related cardiac insufficiency are increasingly recognized [28] and results shown in this study indicate that sacubitril/valsartan can significantly reduce the cardiotoxicity caused by crizotinib.

## 5. Conclusions

Crizotinib induced a range of cardiotoxic side effects in a mouse model, and that increased expression of *Myh7* represents a biomarker for this cardiotoxicity. These cardiovascular abnormalities can be largely prevented by the co-administration of sacubitril/valsartan.

## Data Availability

The datasets used and/or analyzed during the current study are available from the corresponding author on reasonable request.

## Abbreviations

NSCLC, Non-small cell lung cancer; ALK, Anesthenic lymphoma kinase; LV, Left ventricular; RV, Right ventricular; VAs, Ventricular arrhythmias; VRP, Ventricular refractory period; BW, Body weight; HR, Heart rate; BP, Blood pressure; RP, Refractory period; QTc, QT interval correction; GO, Gene ontology; KEGG, Kyoto Encyclopedia of Genes and Genomes; DEGs, Differentially expressed genes; FC, FoldChange; RAS, Renin-angiotensin system; DCM, Dilated cardiomyopathy; PAT, Pulmonary artery acceleration time; LAD, Left atrial diameter; FS, Fractional shortening; LVIDs, Left ventricular diameter at systolic period; LVIDd, Left ventricular diameter at diastolic period; LVAWs, Left ventricular anterior wall thickness at systolic period; LVAWd, Left ventricular anterior wall thickness at diastolic period; LVPWs, Left ventricular posterior wall thickness at systolic period; LVPWd, Left ventricular posterior wall thickness at diastolic period; IVSs, Interventricular septum thickness at systolic period; IVSd, Interventricular septum thickness at diastolic period; SBP, Systolic arterial blood pressure; DBP, Diastolic arterial blood pressure; MBP, Mean arterial blood pressure; HE, hematoxylin and eosin; PVDF, Polyvinylidene fluoride; CV, Conduction velocity; AI, Absolute inhomogeneity; II, Inhomogeneity index; ATP, Adenosine-triphosphate; RT-PCR, Real time-polymerase chain reaction; BCA, Bicinchoninic acid; FDA, Food and Drug Administration; SDS-PAGE, Sodium dodecyl sulfate polyacrylamide gel electrophoresis; LSD, Least significant difference; EF, Ejection fraction; ECG, Electrocardiogram.

## References

[1] Gelatti ACZ, Drilon A, Santini FC (2019). Optimizing the sequencing of tyrosine kinase inhibitors (TKIs) in epidermal growth factor receptor (EGFR) mutation-positive non-small cell lung cancer (NSCLC). *Lung Cancer*.

[2] Planchard D, Popat S, Kerr K, Novello S, Smit EF, Faivre-Finn C (2018). Metastatic non-small cell lung cancer: ESMO Clinical Practice Guidelines for diagnosis, treatment and follow-up. *Annals of Oncology*.

[3] Mao Y, Yang D, He J, Krasna MJ (2016). Epidemiology of Lung Cancer. *Surgical Oncology Clinics of North America*.

[4] Peng L, Zhu L, Sun Y, Stebbing J, Selvaggi G, Zhang Y (2022). Targeting ALK Rearrangements in NSCLC: Current State of the Art. *Frontiers in Oncology*.

[5] Marinelli D, Siringo M, Metro G, Ricciuti B, Gelibter AJ (2022). Non-small-cell lung cancer: how to manage *ALK-, ROS1*- and *NTRK*-rearranged disease. *Drugs in Context*.

[6] Cappuzzo F, Moro-Sibilot D, Gautschi O, Boleti E, Felip E, Groen HJM (2015). Management of crizotinib therapy for ALK-rearranged non-small cell lung carcinoma: an expert consensus. *Lung Cancer*.

[7] Shaw AT, Ou SHI, Bang YJ, Camidge DR, Solomon BJ, Salgia R (2014). Crizotinib in ROS1-rearranged non-small-cell lung cancer. *The New England Journal of Medicine*.

[8] Kwak EL, Bang YJ, Camidge DR, Shaw AT, Solomon B, Maki RG (2010). Anaplastic lymphoma kinase inhibition in non-small-cell lung cancer. *The New England Journal of Medicine*.

[9] Ou SHI, Kwak EL, Siwak-Tapp C, Dy J, Bergethon K, Clark JW (2011). Activity of crizotinib (PF02341066), a dual mesenchymal-epithelial transition (MET) and anaplastic lymphoma kinase (ALK) inhibitor, in a non-small cell lung cancer patient with de novo MET amplification. *Journal of Thoracic Oncology*.

[10] Rothschild SI, Gautschi O (2013). Crizotinib in the treatment of non–small-cell lung cancer. *Clinical Lung Cancer*.

[11] Liu Y, Chen C, Rong C, He X, Chen L (2022). Anaplastic Lymphoma Kinase Tyrosine Kinase Inhibitor-Associated Cardiotoxicity: A Recent Five-Year Pharmacovigilance Study. *Frontiers in Pharmacology*.

[12] Sahu A, Prabhash K, Noronha V, Joshi A, Desai S (2013). Crizotinib: A comprehensive review. *South Asian Journal of Cancer*.

[13] Dai X, Guo G, Zou P, Cui R, Chen W, Chen X (2017). (S)-crizotinib induces apoptosis in human non-small cell lung cancer cells by activating ROS independent of MTH1. *Journal of Experimental & Clinical Cancer Research*.

[14] Andraos E, Dignac J, Meggetto F (2021). NPM-ALK: A Driver of Lymphoma Pathogenesis and a Therapeutic Target. *Cancers*.

[15] Ziogas DC, Tsiara A, Tsironis G, Lykka M, Liontos M, Bamias A (2018). Treating ALK-positive non-small cell lung cancer. *Annals of Translational Medicine*.

[16] Zaborowska-Szmit M, Krzakowski M, Kowalski DM, Szmit S (2020). Cardiovascular Complications of Systemic Therapy in Non-Small-Cell Lung Cancer. *Journal of Clinical Medicine*.

[17] Wang K, Li J, Sun J, Li L, Zhang X, Zhang J (2021). Recommendations from Experts in the Management of Adverse Reactions to ALK Inhibitors (2021 Version). *Zhongguo Fei Ai Za Zhi*.

[18] Gallucci G, Tartarone A, Lombardi L, Aieta M (2015). When crizotinib-induced bradycardia becomes symptomatic: role of concomitant drugs. *Expert Review of Anticancer Therapy*.

[19] Ou SHI, Tong WP, Azada M, Siwak-Tapp C, Dy J, Stiber JA (2013). Heart rate decrease during crizotinib treatment and potential correlation to clinical response. *Cancer*.

[20] Tartarone A, Gallucci G, Lazzari C, Lerose R, Lombardi L, Aieta M (2015). Crizotinib-induced cardiotoxicity: the importance of a proactive monitoring and management. *Future Oncology*.

[21] Zhang Z, Huang TQ, Nepliouev I, Zhang H, Barnett AS, Rosenberg PB (2017). Crizotinib Inhibits Hyperpolarization-activated Cyclic Nucleotide-Gated Channel 4 Activity. *Cardio-oncology*.

[22] Sobczuk P, Czerwińska M, Kleibert M, Cudnoch-Jędrzejewska A (2022). Anthracycline-induced cardiotoxicity and renin-angiotensin-aldosterone system-from molecular mechanisms to therapeutic applications. *Heart Failure Reviews*.

[23] Totzeck M, Schuler M, Stuschke M, Heusch G, Rassaf T (2019). Cardio-oncology - strategies for management of cancer-therapy related cardiovascular disease. *International Journal of Cardiology*.

[24] Sun Y, Song S, Zhang Y, Mo W, Zhang X, Wang N (2022). Effect of angiotensin receptor neprilysin inhibitors on left atrial remodeling and prognosis in heart failure. *ESC Heart Failure*.

[25] Kario K (2018). The Sacubitril/Valsartan, a First-in-Class, Angiotensin Receptor Neprilysin Inhibitor (ARNI): Potential Uses in Hypertension, Heart Failure, and Beyond. *Current Cardiology Reports*.

[26] Gaziano TA, Fonarow GC, Velazquez EJ, Morrow DA, Braunwald E, Solomon SD (2020). Cost-effectiveness of Sacubitril-Valsartan in Hospitalized Patients Who Have Heart Failure With Reduced Ejection Fraction. *JAMA Cardiology*.

[27] Zhang R, Sun X, Li Y, He W, Zhu H, Liu B (2022). The Efficacy and Safety of Sacubitril/Valsartan in Heart Failure Patients: A Review. *Journal of Cardiovascular Pharmacology and Therapeutics*.

[28] Li Y, Kang L, Rong K, Zhang Y, Suo Y, Yuan M (2021). Renal protective effects and mechanisms of the angiotensin receptor-neprilysin inhibitor LCZ696 in mice with cardiorenal syndrome. *Life Sciences*.

[29] Bunsawat K, Ratchford SM, Alpenglow JK, Park SH, Jarrett CL, Stehlik J (2021). Sacubitril-valsartan improves conduit vessel function and functional capacity and reduces inflammation in heart failure with reduced ejection fraction. *Journal of Applied Physiology*.

[30] Li LYF, Lou Q, Liu GZ, Lv JC, Yun FX, Li TK (2020). Sacubitril/valsartan attenuates atrial electrical and structural remodelling in a rabbit model of atrial fibrillation. *European Journal of Pharmacology*.

[31] Wang Y, Tse G, Roever L, Liu T (2020). Sacubitril/valsartan in the treatment of cancer therapy-related cardiac dysfunction. *International Journal of Cardiology*.

[32] Duraes AR, de Souza Lima Bitar Y, Neto MG, Mesquita ET, Chan JS, Tse G (2022). Effectiveness of sacubitril-valsartan in patients with cancer therapy-related cardiac dysfunction: a systematic review of clinical and preclinical studies. *Minerva Medica*.

[33] Martín-Garcia A, López-Fernández T, Mitroi C, Chaparro-Muñoz M, Moliner P, Martin-Garcia AC (2020). Effectiveness of sacubitril-valsartan in cancer patients with heart failure. *ESC Heart Failure*.

[34] Xia Y, Chen Z, Chen A, Fu M, Dong Z, Hu K (2017). LCZ696 improves cardiac function via alleviating Drp1-mediated mitochondrial dysfunction in mice with doxorubicin-induced dilated cardiomyopathy. *Journal of Molecular and Cellular Cardiology*.

[35] Miyoshi T, Nakamura K, Amioka N, Hatipoglu OF, Yonezawa T, Saito Y (2022). LCZ696 ameliorates doxorubicin-induced cardiomyocyte toxicity in rats. *Scientific Reports*.

[36] Dong X, Tse G, Hao G, Du Y (2022). Heterogeneities in Ventricular Conduction Following Treatment with Heptanol: A Multi-Electrode Array Study in Langendorff-Perfused Mouse Hearts. *Life*.

[37] Shi Y, Li Y, Yin J, Hu H, Xue M, Li X (2019). A novel sympathetic neuronal GABAergic signalling system regulates NE release to prevent ventricular arrhythmias after acute myocardial infarction. *Acta Physiologica*.

[38] Park S, Cho EA, Chun JN, Lee DY, Lee S, Kim MY (2022). Crizotinib attenuates cancer metastasis by inhibiting TGFβ signaling in non-small cell lung cancer cells. *Experimental & Molecular Medicine*.

[39] Shaw AT, Kim DW, Nakagawa K, Seto T, Crinó L, Ahn MJ (2013). Crizotinib versus chemotherapy in advanced ALK-positive lung cancer. *The New England Journal of Medicine*.

[40] Solomon BJ, Mok T, Kim DW, Wu YL, Nakagawa K, Mekhail T (2014). First-line crizotinib versus chemotherapy in ALK-positive lung cancer. *The New England Journal of Medicine*.

[41] Oyakawa T, Muraoka N, Iida K, Kusuhara M, Kawamura T, Naito T (2018). Crizotinib-induced simultaneous multiple cardiac toxicities. *Investigational New Drugs*.

[42] Baruch G, Rothschild E, Kapusta L, Schwartz LA, Biner S, Aviram G (2019). Impact of right ventricular dysfunction and end-diastolic pulmonary artery pressure estimated from analysis of tricuspid regurgitant velocity spectrum in patients with preserved ejection fraction. *European Heart Journal. Cardiovascular Imaging*.

[43] Davey PP, Barlow C, Hart G (2000). Prolongation of the QT interval in heart failure occurs at low but not at high heart rates. *Clinical Science*.

[44] Ye S, Zhou HB, Chen Y, Li KQ, Jiang SS, Hao K (2021). Crizotinib changes the metabolic pattern and inhibits ATP production in A549 non-small cell lung cancer cells. *Oncology Letters*.

[45] Hadova K, Mesarosova L, Kralova E, Doka G, Krenek P, Klimas J (2021). The tyrosine kinase inhibitor crizotinib influences blood glucose and mRNA expression of GLUT4 and PPARs in the heart of rats with experimental diabetes. *Canadian Journal of Physiology and Pharmacology*.

[46] Shopp GM, Helson L, Bouchard A, Salvail D, Majeed M (2014). Liposomes ameliorate Crizotinib- and Nilotinib-induced inhibition of the cardiac IKr channel and QTc prolongation. *Anticancer Research*.

[47] Yue P, Xia S, Wu G, Liu L, Zhou K, Liao H (2021). Attenuation of Cardiomyocyte Hypertrophy via Depletion Myh7 using CASAAV. *Cardiovascular Toxicology*.

[48] Yousaf M, Khan WA, Shahzad K, Khan HN, Ali B, Hussain M (2022). Genetic Association of Beta-Myosin Heavy-Chain Gene (MYH7) with Cardiac Dysfunction. *Genes*.

[49] Docherty KF, Vaduganathan M, Solomon SD, McMurray JJV (2020). Sacubitril/Valsartan: Neprilysin Inhibition 5 Years After PARADIGM-HF. *JACC: Heart Failure*.

[50] Singh JSS, Burrell LM, Cherif M, Squire IB, Clark AL, Lang CC (2017). Sacubitril/valsartan: beyond natriuretic peptides. *Heart*.

[51] Sauer AJ, Cole R, Jensen BC, Pal J, Sharma N, Yehya A (2019). Practical guidance on the use of sacubitril/valsartan for heart failure. *Heart Failure Reviews*.

[52] Wei Z, Zhang M, Zhang Q, Gong L, Wang X, Wang Z (2022). A narrative review on sacubitril/valsartan and ventricular arrhythmias. *Medicine*.

[53] Wang R, Ye H, Ma L, Wei J, Wang Y, Zhang X (2022). Effect of Sacubitril/Valsartan on Reducing the Risk of Arrhythmia: A Systematic Review and Meta-Analysis of Randomized Controlled Trials. *Frontiers in Cardiovascular Medicine*.

[54] Kim HM, Kim KH, Park JS, Oh BH (2021). Beneficial Effect of Left Ventricular Remodeling after Early Change of Sacubitril/Valsartan in Patients with Nonischemic Dilated Cardiomyopathy. *Medicina*.

